# Reference Data for Fat Mass and Fat-Free Mass Measured by Bioelectrical Impedance in Croatian Youth

**DOI:** 10.3390/ijerph18168501

**Published:** 2021-08-11

**Authors:** Mario Kasović, Lovro Štefan, Boris Neljak, Vilko Petrić, Damir Knjaz

**Affiliations:** 1 Department of General and Applied Kinesiology, Faculty of Kinesiology, University of Zagreb, 10000 Zagreb, Croatia; mario.kasovic@kif.hr (M.K.); boris.neljak@kif.hr (B.N.); 2 Department of Sports Motorics and Methodology in Kinanthropology, Faculty of Sports Studies, Masaryk University, 62 500 Brno, Czech Republic; 3 Recruitment and Examination (RECETOX), Faculty of Science, Masaryk University, 62 500 Brno, Czech Republic; 4 Department of Education, Faculty of Teacher Education, University of Rijeka, 51000 Rijeka, Croatia; vilko.petric@ufri.uniri.hr; 5 Department of Sports Kinesiology, Faculty of Kinesiology, University of Zagreb, 10000 Zagreb, Croatia; damir.knjaz@kif.hr

**Keywords:** normative, children, adolescents, body composition

## Abstract

Fat mass and fat-free mass have become useful clinical indices in determining healthy growth and physical development during critical periods of childhood and adolescence; however, despite a wide range of nutritional surveillance its study is limited by a lack of reference data. The purpose of this study was to establish sex-specific and age-specific standards for fat mass and fat-free mass in a large sample of Croatian children and adolescents. In this cross-sectional study, we collected data from 12,678 participants aged 11 to 18 years old (mean age ± standard deviation (SD): 14.17 ± 2.25 years; height 164.56 ± 11.31 cm; weight: 57.45 ± 13.73 kg; body mass index: 21.24 ± 3.67 kg/m^2^; 53% girls). Fat mass and fat-free mass were measured three times by bioelectrical impedance. The Lambda, Mu and Sigma methods were used to create percentile charts for fat mass index (FMI) and fat-free mass index (FFMI; fat mass and fat-free mass divided by height^2^). Sex and age differences were calculated using an analysis of variance (ANOVA) with post hoc comparisons. Boys had lower FMI (from 2.66 to 3.89) and higher FFMI values (from 16.90 to 17.80) in all age groups, compared to girls (for FMI from 2.79 to 5.17 and for FFMI from 14.50 to 14.90, *p* < 0.001). In boys, FMI slightly declined until the age of 14, after which an increase from the age of 15 to 18 was observed. In girls, FMI gradually increased from the age of 11 to 18 (*p* < 0.001). In general, FFMI increased by age in boys [F(7,5440) = 52.674, *p* < 0.001], while girls had more stable FFMI across all age groups [F(7,7222) = 2.728, *p* = 0.057]. The newly established sex-specific and age-specific reference data could be used for national surveillance and to screen for children and adolescents with high FMI and low FFMI.

## 1. Introduction

The prevalence of children and adolescents being overweight or has become a public health concern worldwide [1]. Specifically, over the last four decades, mean body mass index in youth has increased in almost every region and country [1]. Measuring fat mass and fat-free mass is an important determinant of nutritional assessment and fitness [2]. Higher levels of fat mass and lower levels of fat-free mass have been consistently associated with adverse health-related outcomes, including metabolic risk [3,4], sarcopenia [5] and all-cause mortality [6]. In youth, excess fat mass [7] and low fat-free mass [8] have become a global public health burden, affecting locomotion and metabolism [9]. For example, a study by Burrows et al. [10] has shown that children with fat-free mass in the lowest quartile (<25th percentile) had a higher risk of metabolic syndrome compared to those with fat-free mass above the 25th percentile.

Despite previous evidence that body weight is a simple tool for assessing nutritional status, this measure cannot discriminate among differences in the proportion of fat mass and fat-free mass [11,12]. Currently, several reliable and valid imaging techniques have been proposed to measure body composition, including dual-energy X-ray absorptiometry, whole–body K^+^ counting, 24 h creatinine excretion [13,14,15], plethysmography [16], multi-slice computerized tomography (CT) [17] and magnetic resonance imaging (MRI) [18]. Unfortunately, the majority of these tools are complex, time-consuming and expensive [19]. Bioelectrical impedance analysis is a safe, portable, inexpensive, simple, rapid and non–invasive method to assess body composition and can distinguish between fat mass and fat-free mass [19,20]. It also calculates body cell mass, total body water, intracellular and extracellular water, and has excellent consistency for repeated measurements. Although this technique provides slightly less accurate data [19], it offers an important practical advantage for measuring body composition in clinical and population-based settings.

Quantifying fat mass and fat-free mass in youth needs to be a priority, since these two tissues have different effects on insulin sensitivity and energy disposal, predicting the risk for metabolic disease more accurately [21]. To be able to classify individuals at higher risk, reference-based standards are needed. However, only a handful of studies have proposed reference data for fat mass and fat-free mass in children and adolescents measured by bioelectrical impedance [2,19,22,23]. The shortcomings in these studies were relatively small sample sizes and region-specific populations of children and adolescents, while a national-based approach has been less studied. For example, two studies by McCarthy et al. [2,19] have been conducted on an opportunistic sample of school children taken from three regions, while no representative approach was used. Additionally, the same group of authors highlighted the importance of geographical, ethnic and nutritional considerations in designing the sampling frame. Similar arguments have been proposed in the studies by Sung et al. [22] and Kurtoglu et al. [23]. Moreover, none of these studies normalized body composition indices by height, the practical implication of which is discussed below.

Therefore, the main purpose of the present study was to establish sex-specific and age-specific standards for fat mass and fat-free mass in a large sample of Croatian children and adolescents. We hypothesized that boys would have lower fat mass and higher fat-free mass in all age groups compared to girls.

## 2. Materials and Methods

### 2.1. Ethics Approval

Before the study began, all the participants and their parents/guardians had given written informed consent for their participation. All procedures were anonymous and followed the Declaration of Helsinki; the study was also approved by the Ethical Committee of the Faculty of Kinesiology and the Agency for Science and Higher Education (ethical code number: 01/2011).

### 2.2. Study Participants

This study was part of the Croatian Fitness (CROFIT) project, a national-based study aiming to establish new normative data for body composition in primary and secondary school children and adolescents aged 11–18 years. The selection process was random and stratified by county. Specifically, Croatia has 21 counties and within each county, 20 schools were randomly selected. This gives a total of four classes and approximately 100 students in each school. The protocol was standardized for primary (ages 11–14) and secondary (ages 15–18) schools within each county. Each school had the same probability of entering the study, by drawing school codes on slips of paper from a box. Such a sample would give a sample size between 12,000 and 16,000 children and adolescents, or between 750 and 1000 students per sex and age group. After the project was completed, the initial sample size was 15,185, of which 2507 did not have body composition indices tested. We based our analyses on 12,678 children and adolescents (≈2.5% of the whole population; 5448 boys and 7230 girls; 53% girls). To justify the sample size, we performed the sample size calculation. Of 464,000 primary and secondary school students, using a 99% confidence level and a 1.13% confidence interval, our final sample would be 12,023. Of note, the percentages of underweight, normal weight, overweight and obesity in the studied sample were 3.1%, 83.4%, 9.4% and 4.1%, respectively, based on the establishment of a standard definition for child body mass index status [24].

### 2.3. Body Composition Analysis

To assess body composition, we used bioelectrical impedance analysis (Omron BF500 Body Composition Monitor, Omron Medizintechnik, Vernon Hills, IL, USA). The device uses eight electrodes and requires the participant to stand on metal footpads barefoot and grasp a pair of electrodes fixed on a handle with arms extended in front of the chest [25]. The manufacturer’s pre-programmed equations were used to predict fat mass and fat-free mass. The participants performed the test three times to assess the level of internal consistency. The reliability coefficient for three measurements in each sex and age group was almost perfect (Cronbach’s alpha > 0.99). The testing procedure took place between 9:00 and 11:00 a.m. and all participants were instructed not to consume food or water before testing. The same equipment was used in each school. Standing height and weight were measured following instructions from previous studies [12] using Seca portable 202 scales (Seca, Hamburg, Germany) and a digital scale (Seca, model 769). Fat mass index (FMI) and fat-free mass index (FFMI) were normalized for height by dividing by height^2^ and the units were expressed in kg/m^2^ [12]. The reason for this approach was that children and adolescents with the same body weight and fat mass but different height will have a different body composition status [12].

### 2.4. Statistical Analysis

Basic descriptive statistics are presented as mean and standard deviations (SD). Kolmogorov–Smirnov tests showed that the data were normally distributed. Differences between boys and girls were calculated with the Student’s *t*-test for independent samples. Sex and age interaction differences were calculated by the analysis of variance (ANOVA) with a post hoc comparison test between the groups. For each variable, we determined sex-specific and age-specific percentile values (2nd, 9th, 25th, 50th, 75th, 91st, and 98th percentile) and used the Lambda (L), Mu (M) and Sigma (S) method, in which the optimal power to obtain normality is summarized by a smooth (L) curve and statistics in the mean (M) and coefficient of variation (S) are similarly smoothed. Next, all three curves (L, M and S) are summarized based on the power of age-specific Box–Cox power transformations for normalizing the data. The LMS method assumes that the data can be normalized by a power transformation and removing the skewness [26]. All analyses were performed in Statistical Package for Social Sciences (SPSS) version 23 (SPSS Inc., Chicago, IL, USA).

## 3. Results

Sex-specific and age-specific descriptive statistics of the study participants are presented in Table 1. In Table 2, it can be seen that boys had lower FMI and higher FFMI values in all age groups compared to girls (*p* < 0.001). In boys, FMI slightly declined until the age of 14, after which an increase from the age of 15 to 18 was observed. In girls, FMI gradually increased from the age of 11 to 18 (*p* < 0.001). In general, FFMI increased by age in boys [F(7,5440) = 52.674, *p* < 0.001], while girls had more stable FFMI across all age groups [F(7,7222) = 2.728, *p* = 0.057]. Of note, boys were taller, heavier and had higher body mass index and fat-free mass levels (both in kg and %) compared to girls (*p* < 0.001).

**Figure 1:**
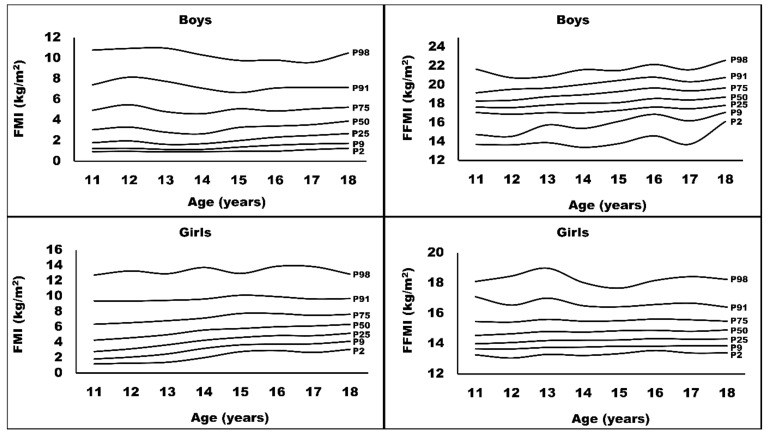
Sex-specific and age-specific percentiles for FMI (fat mass index) and FFMI (fat-free mass index) of the study participants (*n* = 12,678).

Sex-specific and age-specific reference data for FMI and FFMI are presented in Table 2. Boys had lower median values (P50) of FMI [F(1,12,678) = 2008.797, *p* < 0.001] and higher FFMI [F(1,12,678) = 11,655.294, *p* < 0.001] compared to girls. In girls, the increase in FMI across all age groups was more pronounced in all percentiles compared to boys. However, boys experienced a rising statistics in FFMI, with a slightly lower value at the age of 17. The FMI and FFMI percentile curves for boys and girls are presented in Figure 1.

## 4. Discussion

The purpose of this study was to establish sex-specific and age-specific standards for fat mass and fat-free mass in a large sample of Croatian children and adolescents. The main findings are: (1) boys have lower FMI and higher FFMI values in all age groups compared to girls; (2) FMI does not change in boys, while in girls, it gradually increases with age; (3) FFMI increases by age in boys but does not change in girls.

This is the first study providing sex-specific and age-specific reference data for FMI and FFMI in children and adolescents measured by bioelectrical impedance. Only a handful of studies in youth have used bioelectrical impedance to provide reference data for fat mass [19] and fat-free mass [2]. A study by McCarthy et al. [19] has shown that the 50th percentile in boys is relatively stable across age and ranges between 15.0% and 18.0%. The additional analyses showed that the mean value of fat mass percentage in our study was 16.5%, with the highest peak at the age of 12, which is similar to previous evidence [19]. In girls, the same study has shown that fat mass percentage on the 50th percentile continues to increase slightly from 11 to 20 years of age [19]. Additionally, the mean body height values of the aforementioned study were similar to our study and the same patterns of changes and variations in FMI can be expected [19]. Another study aiming to create sex-specific and age-specific reference curves for fat-free mass in boys has shown that the mean value of fat-free mass ranges from 36.4 kg in 11–13-year-olds to 59.7 kg in 17–20-year-olds [2], which is similar to our findings (mean value of fat-free mass: 45.1 kg). In girls, the approximate mean value was 40.0 kg [2], which is confirmed by the results of this study (mean value of fat-free mass: 39.7 kg). Although previous evidence did not normalize the outcome variables by height, we could assume that similar percentile charts could have been obtained. The changes in FMI and FFMI between boys and girls can be discussed through a few biological mechanisms. The proportion of fat-free mass decreases with age in girls, while in boys it remains relatively stable across childhood and early teens, at which point it starts to increase, reflecting the testosterone-driven increase and mineral tissue [27]. On the other hand, the level of body fat increases until puberty, when hormones induce a pronounced sexual dimorphism whereby boys gain more muscle and lean tissue and girls lay down fat mass [2].

Our newly proposed FMI and FFMI values should be used as an addition to body mass index values in White children and adolescents. These reference values are especially relevant because they can detect individuals at increased risk of cardiovascular and metabolic disorders more precisely than body weight or body mass index [12]. It has been highlighted that body composition indices vary geographically [2], which means that previously published reference data in children and adolescents may not be appropriate for classifying our sample. Previous evidence has established clinically and epidemiologically useful cutoffs based on the body mass index cutoffs adopted by the International Obesity Task Force [19]. Specifically, a study by McCarthy et al. [19] recommended cutoffs for defining under fat (2nd percentile), normal fat (<85th percentile), overfat (≥85th–<95th percentile) and obesity (≥95th percentile). It has been recognized that obesity, defined by fat mass, predicts more precisely the level of metabolic disorders than obesity assessed by body mass index [28], increasing the risk of type 2 diabetes, dyslipidemia and raised fasting glucose [28]. On the other hand, higher levels of fat-free mass may help in stabilizing insulin-induced glucose stored in skeletal muscles [29].

This study is not without limitations. First, body composition levels in growing children and adolescents should be obtained from longitudinal studies that give the possibility to assess natural changes in individual growth and development [30]. Second, biological maturation was not measured in this study, diminishing the range of variability between individuals of the same chronological age during adolescence. Third, hydration status has been shown to affect the accuracy of bioelectrical impedance analysis [31]. Although the participants were instructed before the measurement not to drink or eat, we cannot exclude the possibility of procedure–error. Nevertheless, all the participants enrolled in the study were healthy, assuming a stable water balance. Fourth, previous evidence has highlighted the importance of additional conditions of the measurements for different ages, i.e., the activity of the cells gives rise to a different dependence on electrical frequency [32]. Specifically, a study by Martines-Arano et al. [32] has shown that electrical sensing of the cell may have different aging absorption and by using bioelectrical impedance, additional cell malfunctions can be observed. Finally, we did not assess outside nor skin temperature, which change resistance measurements of the bioelectrical impedance. For example, fat mass has been shown to decrease in warm, compared to cool conditions [33].

## 5. Conclusions

This study shows sex-specific and age-specific percentile curves for FMI and FFMI in a large sample of Croatian children and adolescents. Specifically, boys have lower FMI and higher FFMI values in all age groups, compared to girls and different statistics across the age groups between sexes is observed; the FMI values remain unchanged in boys, but increase in girls, yet the FFMI values increase in boys and stagnate in girls. By using our reference data, health-related professionals may be able to detect those individuals with higher fat mass and lower fat-free mass levels. Second, our baseline results can be used to observe secular and longitudinal percentile changes and group those individuals with the largest negative changes (higher levels of fat mass and lower levels of fat-free mass) into specific clusters for special interventions and policies. Finally, school-based settings and physical education classes can be an easy means of promoting higher physical activity and physical fitness, healthy dietary choices and less time adopting sedentary behaviors to decrease the level of fat mass and increase fat-free mass. Future research aiming to develop normative data for different body composition indices should be using more objective methods in a longitudinal design to establish biological changes of fat mass and fat-free mass from childhood to adulthood.

## Figures and Tables

**Table 1:** Sex-specific descriptive statistics of the study participants (*n* = 12,678).

Study Variables	Total Sample (*n* = 12,678)	Boys (*n* = 5448)	Girls (*n* = 7230)	*p*-Value *
	Mean ± SD	Mean ± SD	Mean ± SD	
Age (years)	14.17 ± 2.30	14.11 ± 2.22	14.23 ± 2.28	<0.001
Height (cm)	164.56 ± 11.31	167.13 ± 13.46	162.30 ± 8.37	<0.001
Weight (kg)	57.45 ± 13.73	59.91 ± 15.69	55.28 ± 11.31	<0.001
Body mass index (kg/m^2^)	21.24 ± 3.67	21.72 ± 3.71	20.88 ± 3.61	<0.001
Fat mass (kg)	13.72 ± 7.68	11.10 ± 7.07	15.71 ± 7.52	<0.001
Fat mass (%)	22.54 ± 9.63	16.54 ± 7.88	27.05 ± 8.28	<0.001
FMI (kg/m^2^)	5.00 ± 2.77	3.82 ± 2.29	5.89 ± 2.71	<0.001
Fat-free mass (kg)	45.10 ± 9.90	52.23 ± 10.11	39.73 ± 5.29	<0.001
Fat-free mass (%)	77.46 ± 9.64	83.46 ± 7.88	72.94 ± 8.28	<0.001
FFMI (kg/m^2^)	16.31 ± 2.16	18.04 ± 1.80	15.02 ± 1.35	<0.001

* denotes calculating sex differences using Student’s *t*-test for independent samples; FMI—fat mass index; FFMI—fat-free mass index; *p* < 0.05.

**Table 2:** Sex-specific and age-specific reference data for FMI and FFMI of the study participants (*n* = 12,678).

Measure	Sex	Age	N	P2	P9	P25	P50	P75	P91	P98
FMI (kg/m^2^) *	Boys	11	634	0.94	1.23	1.80	3.06	4.95	7.42	10.80
		12	764	0.96	1.26	1.97	3.31	5.48	8.15	10.90
		13	842	0.92	1.15	1.64	2.82	4.81	7.75	11.00
		14	852	0.94	1.15	1.70	2.66	4.61	7.09	10.30
		15	555	0.96	1.37	2.00	3.28	5.09	6.66	9.78
		16	562	0.98	1.56	2.33	3.41	4.87	7.10	9.81
		17	613	1.14	1.68	2.50	3.55	5.08	7.15	9.58
		18	626	1.26	1.74	2.68	3.89	5.24	7.16	10.50
	Girls	11	953	1.20	1.83	2.79	4.28	6.35	9.38	12.80
		12	1038	1.29	2.08	3.15	4.59	6.55	9.37	13.30
		13	1203	1.41	2.49	3.67	4.99	6.81	9.46	12.90
		14	1017	2.00	3.21	4.26	5.59	7.15	9.63	13.70
		15	722	2.78	3.67	4.63	5.79	7.75	10.1	13.00
		16	690	2.94	3.76	4.87	6.02	7.74	9.94	13.90
		17	810	2.68	3.81	4.86	6.13	7.50	9.63	13.80
		18	797	3.08	4.13	5.17	6.33	7.64	9.69	12.90
FFMI (kg/m^2^) **	Boys	11	634	13.70	14.70	17.10	17.60	18.30	19.20	21.70
		12	764	13.70	14.60	16.90	17.60	18.40	19.50	20.80
		13	842	13.90	15.80	17.10	17.90	18.80	19.70	20.90
		14	852	13.40	15.40	17.00	18.10	19.00	20.00	21.60
		15	555	13.80	16.10	17.30	18.10	19.30	20.50	21.50
		16	562	14.60	16.90	17.60	18.60	19.70	20.80	22.20
		17	613	13.70	16.20	17.50	18.40	19.40	20.30	21.60
		18	626	16.10	17.10	17.80	18.70	19.70	20.80	22.60
	Girls	11	953	13.30	13.70	14.00	14.50	15.50	17.10	18.10
		12	1038	13.10	13.70	14.10	14.70	15.40	16.50	18.50
		13	1203	13.30	13.80	14.20	14.80	15.60	17.00	19.00
		14	1017	13.20	13.80	14.20	14.80	15.50	16.50	18.00
		15	722	13.30	13.80	14.20	14.90	15.50	16.40	17.70
		16	690	13.60	13.80	14.30	14.90	15.60	16.60	18.20
		17	810	13.40	13.90	14.30	14.80	15.60	16.70	18.40
		18	797	13.40	13.90	14.30	14.90	15.50	16.40	18.20

* FMI—fat mass, ** FFMI—fat-free mass index.

## Data Availability

No new data were created or analyzed in this study. Data sharing is not applicable to this article.
